# 1,25OH-Vitamin D3 and IL-17 Inhibition Modulate Pro-Fibrotic Cytokines Production in Peripheral Blood Mononuclear Cells of Patients with Systemic Sclerosis

**DOI:** 10.7150/ijms.70984

**Published:** 2022-05-09

**Authors:** Addolorata Corrado, Cinzia Rotondo, Eliana Rita Sanpaolo, Alberto Altomare, Nicola Maruotti, Daniela Cici, Francesco Paolo Cantatore

**Affiliations:** Rheumatology Clinic - Department of Medical and Surgical Sciences, University of Foggia, Foggia- Italy

**Keywords:** Systemic Sclerosis, T-lymphocytes, Interleukins, Fibrosis

## Abstract

**Objectives:** IL-17 modulates the synthesis of several molecules involved in the pathogenesis of Systemic Sclerosis (SSc). Vitamin D (1,25(OH)_2_D3) shows anti-fibrotic properties and it is able to affect the IL-17 production in several experimental conditions.

The aim of this study is to assess the production of IL-17A and pro-fibrotic cytokines in peripheral blood mononuclear cells (PBMCs) from subjects with SSc in basal conditions and after treatment with 1,25(OH)2D3 and IL-17A neutralizing antibodies.

**Methods:** The production of IL-17A and pro-fibrotic cytokines (TGFβ, CTGF and FGF2) in PBMCs obtained from 51 SSc patients and 31 healthy subjects was assessed both in basal conditions and in presence of anti-IL17A antibodies and several concentrations of 1,25(OH)_2_D3. The association of cytokines production with clinical disease characteristics and the in vitro effect of 1,25(OH)_2_D3 and IL-17A inhibition were assessed.

**Results:** PBMCs from SSc subjects produced higher amount IL-17A, TGFβ, CTGF and FGF2 compared to healthy controls. IL17, TGFβ, CTGF and FGF2 levels were higher in SSc patients with interstitial lung disease and digital ulcers, whereas IL-17A production was lower in patients with PAH. IL- 17A inhibition reduced the production of FGF2, whereas enhanced the synthesis of TGFβ and CTGF. 1,25(OH)_2_D3 decreased the production of IL17A and pro-fibrotic cytokines in a dose- dependent manner.

**Conclusions:** IL-17A is involved in the regulation of fibrogenesis in SSc, and could represent an intriguing potential therapeutic target, even if its role remains controversial. 1,25(OH)_2_D3 inhibits both IL-17A and pro-fibrotic cytokines, confirming its potential anti-fibrotic effect.

## Introduction

Systemic sclerosis (SSc) is an autoimmune connective tissue disease characterized by vasculopathy and progressive fibrosis of skin and internal organs (gastrointestinal tract, heart, kidneys and lungs) 1, whose pathogenesis involves three main aspects represented by immunological dysregulation, fibrosis and vascular impairment 2. The abnormal immune response plays an essential role in the physio-pathological events that lead to vascular changes and progressive tissue fibrosis, and several evidences confirm that immune cells are among the main effectors that contribute in initiating and maintaining the complex process that culminates in fibrosis and vascular damage. T cells may activate fibroblasts both directly, through cell-cell contact, or indirectly by the effects of secreted cytokines. Both Th1 and Th2 cells are involved in SSc pathogenesis; while the pro-inflammatory Th1 cytokines mediate the inflammatory processes which predominate in the early stage of disease, the Th2 cytokines are mainly involved in the fibrotic processes, by stimulating the production of pro-fibrotic factors 3. Th17 cells are involved in the pathogenesis of several rheumatic autoimmune diseases, such as rheumatoid arthritis, systemic lupus erythematosus, Sjogren syndrome, psoriatic arthritis 4; their role in the pathogenesis of SSc has recently emerged 5 as it has been reported that IL-17, which is the main cytokine secreted by Th17 cells, is overproduced by T cells from the peripheral blood and in fibrotic lesions of the skin and lungs in SSc patients and it is able to enhance the proliferation of fibroblasts *in vitro* 6. IL-17 family includes several isoforms (IL-17A through IL17F), whose most studied member is IL-17A, but the data available about its role in SSc are controversial 7,8. Beyond its direct pro-fibrotic effect, IL-17A is able to modulate the synthesis of several molecules that play a central role in the fibrotic processes, such as TGFβ , CTGF and MMP-1 9-11. In SSc an increased production of pro-inflammatory and pro- fibrotic cytokines is observed, particularly TGFβ, IL-1 and IL-6, which are also linked to Th17 differentiation; this leads to hypothesize that SSc pathogenesis may be related to a polarization of the immune response toward the Th17 pathway 12, and to suggest IL-17 as a possible therapeutic target. To date there are no effective disease-modifying drugs for SSc treatment; in the last years, among various emerging treatments, also the anti-inflammatory, anti-apoptotic, and anti-fibrotic effects of Vitamin D have gained considerable attention and it has been hypothesized that Vitamin D might have a modulating effect on pathogenic processes underlying SSc. Previous studies have shown that 1,25(OH)_2_VitaminD3 (1,25(OH)_2_D3) represses the expression of several cytokines, including IL-17 13 produced by immune cells, confirming a potential immune-modulatory effect. The aim of this study was to explore the potential involvement of IL-17A in the pathogenesis of SSc by the analysis of the *in vitro* expression of IL-17A and pro-fibrotic cytokines in peripheral blood mononuclear cells (PBMCs) from subjects with SSc and the effects of IL-17A neutralizing antibodies. In addition, we also evaluated the effect of 1,25(OH)_2_D3 on the expression of both IL- 17A and pro-fibrotic cytokines.

## Patients and methods

### Patients

51 SSc patients fulfilling the ACR/EULAR 2013 diagnostic criteria for SSc 14 were enrolled in the study. As control group, 31 healthy subjects, matched for age and sex, were selected.

In SSc patients, the modified Rodnan skin score (mRSS) 15, the presence/absence of Raynaud phenomenon, digital ulcers (DU), telangiectasia and calcinosis were evaluated, as well as the presence of extra-pulmonary clinical manifestations (lung functional tests with CO diffusing capacity measurement, chest x-ray, pulmonary high resolution computed tomography, electrocardiogram, echocardiogram). The diagnosis of pulmonary arterial hypertension (PAH) was performed by right heart catheterization (RHC) and defined as mean pulmonary arterial pressure >20 mm Hg with pulmonary artery wedge pressure (PAWP) of ≤15mmHg and increased pulmonary vascular resistance (PVR) ≥ 3WU. Laboratory tests were performed to detect anti- nuclear antibodies (ANA), anti-centromeric antibodies (ACAs) and anti-Extractable Nuclear Antigens (anti-ENA), including anti-Topoisomerase I (anti Scl-70) autoantibodies, C3 and C4 complement fractions, full blood count and renal function 1,2. To avoid any interference of immunosuppressive drugs on leukocytes metabolism, patients who had taken any immunosuppressive therapy for 6 months before blood samples collection were excluded from the study. All recruited subjects stopped Vitamin D supplementation 4 months before blood samples collection.

Appropriate informed consent was obtained from each patient and the study was approved by the Institutional Ethics Committee.

### PBMCs isolation

PBMCs, consisting of lymphocytes and monocytes, were separated from erythrocytes by density centrifugation on a Lymphosep™ (Biowest, Riverside, CA, USA) gradient. After washing, human mononuclear cells were counted and seeded in multiwell plates in the appropriate culture medium, consisting of DMEM high glucose (Corning, Corning, NY, USA) supplemented with antibiotics and fungizone (Gibco-ThermoFisher Scientific, Waltham, MA, USA) and with 2% foetal calf serum and incubated at 37°C in a water-saturated atmosphere with 5% CO_2_. After three hours, cells were resuspended in culture media and transferred in a 48-well plated (1×10^6^ cells/well) and incubated at 37°C in a water-saturated atmosphere with 5% CO_2_, in presence or absence of 1,25(OH)_2_D3 (R&D Systems) at various concentrations (10^-9^ M, 10^-8^ M, 10^-7^M ) and anti-IL-17A antibodies Secukinumab - (Novartis, Basel, Switzerland) 0,3 μM as previously described 16. Cells were stimulated with anti- ImmunoCult^TM^ Human CD3/CD28 T Cell Activator (Stemcell, Vancouver, BC, Canada), as previously described 17,18. After 48 hours the culture supernatants were collected and stored at -20°C for subsequent measurement of IL-17A, TGFβ, FGF-2, CTGF by specific enzyme-linked immunosorbent assay; PBMCs mRNA was extracted and stored at -80°C for real-time PCR analysis as described below.

### Cytokines quantification

To evaluate the release of specific growth factors and cytokines in the cell culture supernatants, the levels of TGFβ, FGF2, CTGF and IL-17A were measured using an enzyme-linked immunosorbent assay kit (TGFβ, FGF2, CTGF Human ELISA Kit, Invitrogen- ThermoFisher Scientific, Waltham, MA, USA and IL-17A Human ELISA Kit, Sigma-Aldrich, St. Louis, MO, USA) both in basal conditions and in presence of anti-IL-17A antibodies and various concentrations of 1,25(OH)_2_D3. Cell-cultures supernatant samples and standards were added to the plates pre-coated with the specific antibodies and incubated according to the manufacturer's instructions. The no-bound proteins were removed and a biotinylated antibody was added. In the second step, the plates were washed and streptavidin-horseradish peroxidase (HRP) was added and incubated. The last washing was performed and 3,3′,5,5′-tetramethylbenzidine (TMB) was added as substrate of HRP. Subsequently stop solution was added and absorbance was measured at 450 nm using a microplate ELISA reader.

Each sample was tested in duplicate and results were expressed as pg/ml.

### RNA isolation and real-time PCR analysis

Total RNA was collected in a Lysing Reagent and extracted using a Total RNA Purification Kit (Norgen Biotek Corp. Thorold, ON, Canada), following the manufacturer's protocol for cultured cells. RNA concentration and purity were measured by the 260 and 280 nm absorbance ratio. To generate cDNA template, 1 μg of total RNA was reverse transcribed with a SuperScript IV VILO Master Mix (Invitrogen-ThermoFisher Scientific, Waltham, MA, USA), performed on a Mastercycler Epgradient thermocycler. Real-time PCR (qPCR) was carried out with Applied Biosystems 7300 Real-Time PCR System. Real-time reactions were prepared with TaqMan fast Advanced Master Mix (Applied Biosystem-ThermoFisher Scientific, Waltham, MA, USA) and the expression of FGF2, CTGF, TGFβ and IL-17A (using primers Hs00266645_m1, Hs00174872_m1, Hs00998133_m1 and Hs00174383_m1 respectively; Applied Biosystem-ThermoFisher Scientific, Waltham, MA, USA) was normalized to housekeeping gene GAPDH (Hs02786624_g1, Applied Biosystem-ThermoFisher Scientific, Waltham, MA, USA). The relative mRNA expression levels were calculated using the comparative method Ct (cycle-threshold fluorescence values). Results were expressed as relative mRNA expression (fold change) over reference sample (healthy untreated cells).

### Statistical analysis

Results were expressed as mean ± SD. The normal distribution was assessed using the Shapiro-Wilk test. Comparisons between the different experimental conditions were assessed by repeated- measures analysis of variance (ANOVA) or Friedman's test as appropriate. Comparisons between healthy controls and SSc patients, and among SSc patient subgroups with different organ involvements, were evaluated by the Student's t-test or Mann- Whitney U-test as appropriate. Correlation between levels of different cytokines was assessed using the Pearson test. The associations between the different cytokines and clinical characteristics were evaluated by means linear regression analysis. Values of p<0,05 were considered as significant.

## Results

### Clinical and demographic characteristics

Healthy controls had a mean age of 53,12 ± 13,08 years, range 24-76; 27 (87%) of them where women and 4 (13%) were men; SSc patients mean age was 57,64±11,75, range 24-76; 47 (92,1%) were women and 4 (7,9%) were men. The mean disease duration (from the first non-Raynaud symptom) at study time was 14,58±8,97 years.

Clinical and demographic characteristic of recruited subjects were illustrated in **Table 1.**

### IL17A, TGFβ, FGF2 and CTGF synthesis and expression by PBMCs in healthy and SSc subjects

The production of IL-17A, FGF2, TGFβ and CTGF was evaluated in anti-CD3/CD28 stimulated PBMCs from healthy donors and SSc patients. The synthesis of IL-17A by PBMCs derived from SSc patients was significantly higher compared to cells derived from healthy subjects (464.5±139.05 pg/ml vs 150±57.3 pg/ml). Compared to healthy subjects, SSc derived PBMCs also showed a greater production of FGF2 (15.77±5.18 pg/ml vs 8.9±1.5 pg/ml), TGFβ (784.1±239.6 pg/ml vs 663.7±84.9) and CTGF (105.2±14.1 pg/ml vs 80.1±11.4 pg/ml) (**Table 2**).

Similarly, SSc derived PBMCs showed a significant increase of mRNA expression of IL-17A and pro-fibrotic cytokines FGF2, TGFβ, CTGF compared to healthy subjects to 2.5-fold, 2.38-fold, 1.4- fold, 1.62-fold (p<0.01) relative mRNA, respectively. At baseline, IL-17A synthesis in PBMCs from SSc patients showed a significant positive correlation with the synthesis of FGF2 (r^2^ 0.683, p<0.001), whereas no correlation with the synthesis of TFGβ and CTGF was observed (r^2^ -0,299, p=0,147 and r^2^ -0.151, p=0.433, respectively). In healthy subjects no correlation between IL-17A production and FGF2 (r^2^ 0.028 p=0.88), TFGβ (r^2^ 0.096, p=0.606) and CTGF (r^2^ 0.349, p=0.06) was found.

In SSc patients, after IL-17A blockade, the positive relationship between IL-17A and FGF2 was still observed (r^2^ 0.683, p=0.001), while IL-17A concentration showed a negative correlation with TGFβ (r^2^ -0.576, p<0.001) and CTGF (r^2^ -0.624, p<0.001).

### Relationship between cytokine production by PBMCs from SSc subjects and clinical manifestations

By univariate analysis the clinical manifestations associated to IL-17A, TGFβ, FGF2 and CTGF production by PBMCs were interstitial lung disease (ILD), PAH and DU (p<0,01).

IL-17A production was significantly higher in SSc patients with ILD compared to patients without ILD (534.130±132.8 vs 396.9±112.5 pg/ml, p<0.0001) and in patients with DU compared to patients without DU (506.4±135 vs 387.7±111, p<0.003); conversely, in patients with PAH, IL-17A levels were found to be lower compared to patients without PAH (392.3±69.5 vs 476±144.3, p=0.026). Patients with ILD showed higher levels of TGFβ, FGF2 and CTGF in comparison to patients without ILD (866.2±253.6 vs 705.1±199.6 pg/ml p=0.015; 18.5±4.7 vs 13±4.1 pg/ml p=0.0001; 111.2±13 vs 99.3±12.8 pg/ml, p=0.002 respectively), same as patients with DU (847.9±236 vs 667.1±204 pg/ml p=0,009; 17.6±4.3 vs 12.3±4.8 pg/ml, p=0.0001; 108.2±13.6 vs 99.6±13.6 pg/ml, p=0,036 respectively). No difference was observed in TGFβ, FGF2 and CTGF production between patients with and without PAH.

### Effects of IL-17A neutralizing antibodies and 25(OH)_2_D3 on pro-fibrotic cytokines

**Figure 1:**
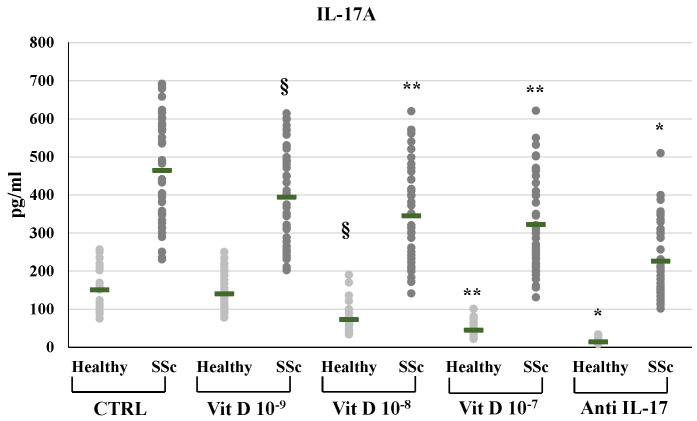
IL-17A synthesis by peripheral blood mononuclear cells (PBMCs) from healthy subjects and SSc subjects in basal conditions (CTRL) and after treatment with several concentrations of 1,25(OH)_2_D3 (10^-9^M, 10^-8^M, 10^-7^M) and IL-17A neutralizing antibodies. IL-17A production is significantly higher in SSc patients compared to healthy controls (p <0.0001) under all experimental conditions. 1,25(OH)_2_D3 significantly decreases IL-17A production in a dose dependent manner both in healthy and in SSc patients (§p<0,05 vs baseline; **p<0,005 vs 10^-9^M). As expected, IL-17A detection is significantly lower in PBMCs treated with anti-IL-17A neutralizing antibodies (*p<0,0001 vs control) both in SSc and healthy PBMCs.

**Figure 2:**
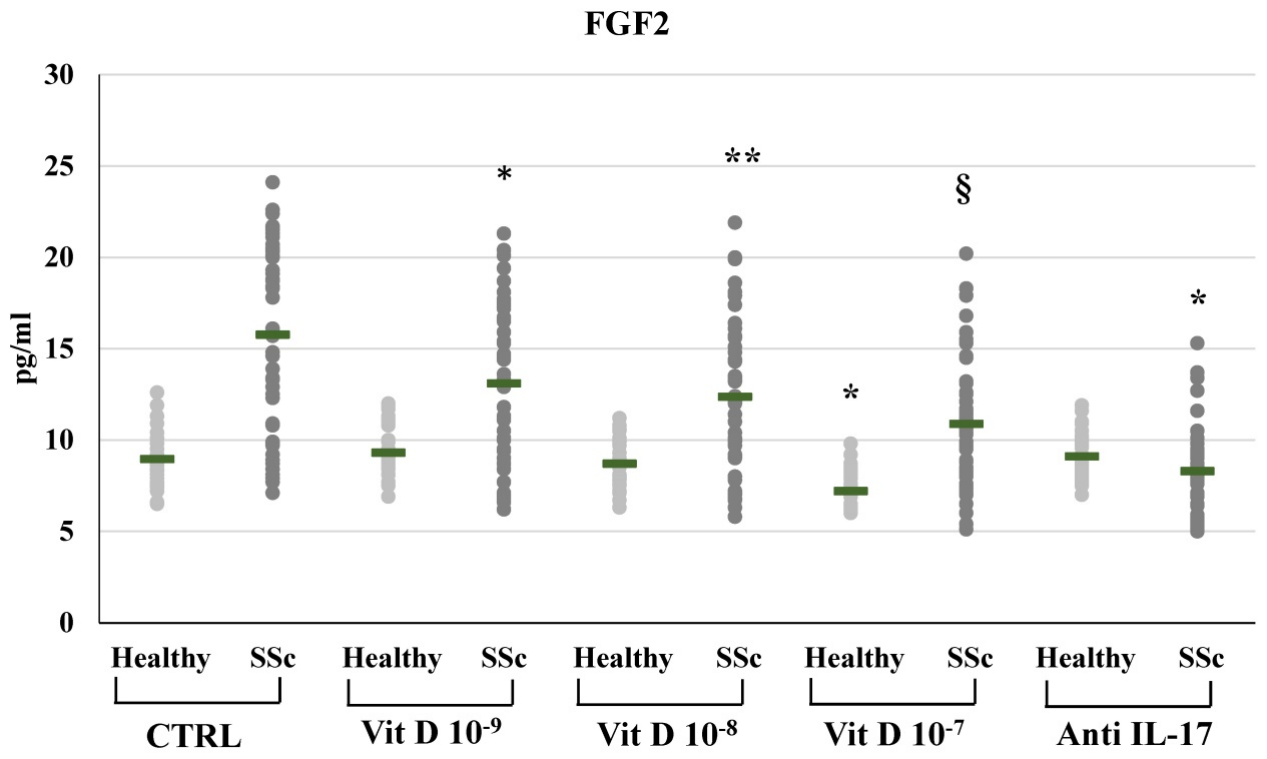
FGF2 synthesis by peripheral blood mononuclear cells (PBMCs) from healthy donors and SSc subjects in basal conditions (CTRL) and after treatment with several concentrations of 1,25(OH)2D3 (10^-9^M, D 10^-8^M, D 10^-7^M) and IL-17A neutralizing antibodies. In PBMCs derived from SSc subjects the FGF2 production is significantly reduced by 1,25(OH)_2_D3 in a dose-dependent manner (*p<0,05 vs baseline; **p<0,05 vs D 10^-9^M; §p<0,05 vs D 10^-8^M). In PBMCs derived from healthy donors, only higher concentration of 1,25(OH)_2_D3 (10^-7^M) induces a significant reduction of FGF2 (*p<0,05 vs baseline). IL-17A neutralizing antibodies significantly reduces FGF2 production only in SSc subjects (*p<0,0001 vs baseline).

**Figure 3:**
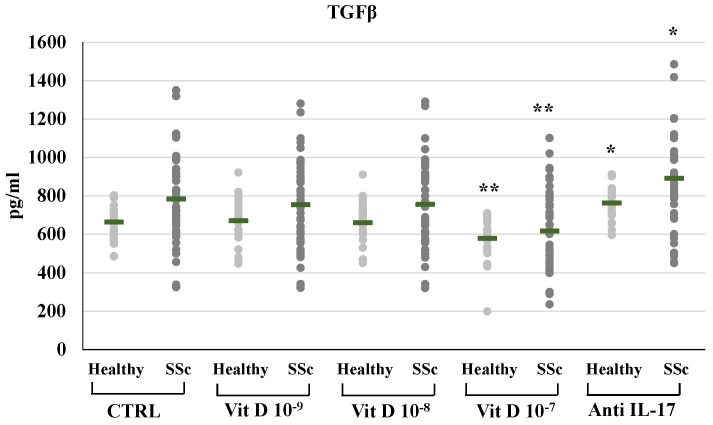
TGFβ synthesis by peripheral blood mononuclear cells (PBMCs) from healthy donors and SSc subjects in basal conditions (CTRL) and after treatment with several concentrations of 1,25(OH)_2_D3 (10^-9^M, D 10^-8^M, D 10^-7^M) and IL-17A neutralizing antibodies. 1,25(OH)_2_D3 induces a significant reduction of TGFβ production in PBMCs cultures only at the highest concentration (10^-7^M) both in healthy and SSc subjects (**p<0,01 vs baseline). Conversely, IL-17A neutralizing antibodies significantly increases TGFβ production both in healthy and SSc subjects (*p<0,01 vs baseline).

**Figure 4:**
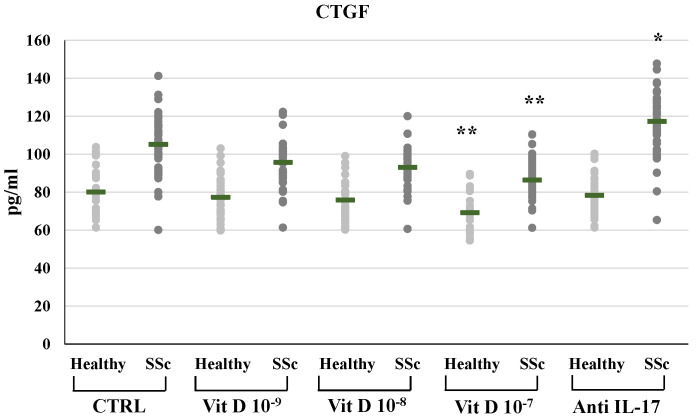
CTGF synthesis by peripheral blood mononuclear cells (PBMCs) from healthy donors and SSc subjects in basal conditions (CTRL) and after treatment with several concentrations of 1,25(OH)_2_D3 (10^-9^M, D 10^-8^M, D 10^-7^M) and IL-17A neutralizing antibodies. 1,25(OH)_2_D3 significantly reduces CTGF synthesis in PBMCs from SSc patients only at the highest concentration both in SSc and healthy subjects (10-7 M **p<0,05 vs baseline). Conversely, IL-17A neutralizing antibodies significantly enhanced CTGF production, but only in PBMCs derived from SSc subjects (*p<0,001 vs baseline).

In SSc derived PBMCs, neutralizing IL-17A antibodies significantly decreased the synthesis of FGF2 (8.29±2.3 vs 15.77±5.18 pg/ml) and relative mRNA levels (from 2,4-fold to 0,89-fold compared to untreated control, p<0.01); conversely IL-17A inhibition significantly enhanced the synthesis and mRNA expression of TFGβ (891.53±22.5 vs 784.12±239.6 pg/ml p<0.01, relative mRNA levels from 1.4-fold to 1.9-fold, p<0.05) and CTGF (117.29±16.6 vs 105.2±14.1 pg/ml, relative mRNA levels from 1.62-fold to 2.01-fold, p<0.05). On the other hand, in healthy donors derived PBMCs, the inhibition of IL-17A did not affect the synthesis and mRNA expression of FGF2 (9.1±1.3 vs 8.9±1.5 pg/ml; 0.9-fold relative mRNA) and CTGF (78.4±10.3 vs 80.1±11.4 pg/ml; 1.07-fold relative mRNA) whereas increased TFGβ (763.4±84.9 vs 663.73±84.8 pg/ml; 1,2- fold relative mRNA) **(Figures 1, 2, 3, 4)**. As expected, treatment with IL-17A neutralizing antibodies reduced the detection of IL-17A in the supernatant cell cultures (13.77±6.04 vs 150.85±57.4 pg/ml in healthy subjects, p<0.001; 226.43±94.03 vs 464.54±139.5 pg/ml in SSc patients, p<0.001) whereas it did not affect IL-17A mRNA expression (relative mRNA levels 0.9- fold in healthy subjects; from 2.5-fold to 2.38-fold in SSc compared to control).

In SSc derived PBMCs, treatment with 1,25(OH)_2_D3 induced a significant reduction of FGF2 synthesis compared to untreated cells in a dose-dependent manner (10^-9^M 13.09±4.4; 10^-8^M 12.36±4.06; 10^-7^M 10.87±3.53 vs 15.76±5.18 pg/ml in untreated cells) **(Figure 2)**. Also FGF2 mRNA relative expression was reduced in 1,25(OH)_2_D3 treated cells in a dose-dependent manner (from 2.38-fold in SSc untreated cells to 2-fold in 10^-9^M, p<0.05; to 1.75-fold in 10^-8^M p<0.01; to 1.3-fold in 10^-7^M, p<0.001 compared to control). In healthy subjects, only the highest 1,25(OH)_2_D3 concentration reduced the synthesis and the production of FGF2 (8.95±11.5 vs 7.19±1.02 pg/ml, p<0.05; 0.58-fold relative mRNA expression, p<0.01).

1,25(OH)_2_D3 also induced a significant reduction of TFGβ synthesis and mRNA expression in SSc patients, but only at the highest concentration (10^-7^M: 616.8±202.3 vs 784.11±239.6 pg/ml, **Figure 3**; relative mRNA levels from 1.4-fold in untreated cell to 0.80, p<0.01 compared to control); similarly, in PBMCs from healthy subjects, treatment with the highest concentration of 1,25(OH)_2_D3 significantly reduced TGFβ synthesis and mRNA expression (10^-7^M: 578.91±101.4 vs 663.75±84.9 pg/ml, p<0.05, **Figure 3**; relative mRNA levels 0,80-fold compared to control, p<0.05).

CTGF synthesis and expression were significantly reduced in SSc derived PBMCs treated with 1,25(OH)_2_D3 only at the highest concentration (10^-7^M: 86.41±10.11 vs 105.2±14.1 pg/ml in untreated cells, **Figure 4**; mRNA relative expression from 1.62-fold in untreated cells to 1.22 p<0.05 compared to control), similarly to the reduction of CTGF observed in PBMCs from healthy subjects only at the highest 1,25(OH)_2_D3 concentration (10^-7^M: 69.2±9.1 vs 80.1±11.47 pg/ml in untreated cells, **Figure 4**; mRNA relative expression 0.53-fold p<0.01).

The addition of 1,25(OH)_2_D3 in PBMCs significantly reduced the production and the mRNA expression of IL-17A, in a dose-dependent way in SSc subjects (10^-9^M: 394.99±126.64, p<0.05; 10^-8^M: 345.44±125.48, p<0.05; 10^-7^M 322.62±123.06, p<0.01 vs 464.54 pg/ml in untreated cells) and in healthy subjects (10^-9^M: 140.54±50.66, n.s.; 10^-8^M 72.55±39.04, p <0,0001; 10^-7^M 45.06±19.5, p<0.0001 vs 150.85±57.38 pg/ml in untreated cells) **(Figure 1)**. IL-17A mRNA expression was reduced in SSc-derived PBMCs treated with 1,25(OH)_2_D3 10^-9^M, 10^-8^M and 10^-7^M from 2.5-fold (untreated cells) to 2-fold (p<0.05), 1.8-fold (p<0.01), 1.51-fold (p<0.001) compared to control and to 0.82-fold (n.s.), 0.5-fold (p<0,001), 0.3-fold (p<0,0001) in PBMCs from healthy subjects, respectively.

## Discussion

The physiopathology of SSc is very complex and not yet completely understood, involving a condition of immune dysregulation and inflammation, in which several cell types, cytokines and growth factors act in a framework of complex interactions; particularly, lymphocytes and monocytes produce a wide range of cytokines which induce vascular changes and tissue fibrotic damage 19. Especially, the Th1/Th2-cell immune response plays a pivotal role in the development of the disease, but in the last years it has been shown that the Th17-related cytokines participate in the pathogenesis of SSc, modulating several inflammatory and fibrotic responses in the context of an intricate mediators and immune cells relationship.

Notably, the role of IL-17 has been proven in several pre-clinical experimental model of cardiac, renal, peritoneal and liver fibrosis 20-23. IL-17 also appears to be directly involved in the pathogenetic events leading to lung fibrosis 10 and it is well known that IL-17 can regulate the release of cytokines involved in fibrosis development, such as CTGF, TGFβ and ICAM-1 in several tissues 24,25.

Nevertheless, there is conflicting evidence concerning the exact function of IL-17 in the fibrotic response in SSc patients. First of all, the isoform of IL-17 which appears to be related to various immunopathogenic aspects of SSc is mainly IL-17A. Several studies described increased levels of IL-17A in serum of SSc patients 26, whereas other reports do not confirm this finding. These inconsistent data may be partly attributed to the difficulty of measuring IL-17A systemically, as the circulating levels are really low, as well as the circulating levels of other cytokines which are often below the threshold values detected by the commonly used ELISA kits, whereas the cytokines detection at cellular or tissue level can be considered more appropriate. Increased mRNA expression and protein synthesis of IL-17A have been reported in skin biopsies from SSc patients 11 and IL-17A is significantly higher in SSc skin biopsies compared to skin biopsies derived from patients with morphea 27. In the skin and lung tissue of the murine model of bleomicin- induced scleroderma, an increased recruitment of Th17 cell and higher IL-17 levels have been reported 28, and the IL-17 blockade reduces the severity of fibrosis 9. Further, in IL-17 knockout mice the severity of lung and skin fibrosis mediated by IL-1 is reduced 8. Human studies also evidence toward a pro-fibrotic effect of IL-17, showing that circulating Th17 cells correlate with SSc activity, and supernatant from Th17 cultured cells stimulates type I collagen synthesis in fibroblasts isolated from the skin of SSc patients 29. Furthermore, it is well known that IL-17 can also increase the release of cytokines involved in fibrosis development, such as CTGF, TGFβ and ICAM-1 in several tissues 24,25. Nevertheless, even if there are many evidences of increased Th17 cell and IL-17A levels in peripheral blood, skin and lung in SSc, and it has been shown that SSc fibroblasts express high amount of IL-17 receptors 30, contrasting results about the effect of IL-17A on pro-fibrotic cytokines in SSc are available, with several data from human studies and experimental studies that argue toward an anti-fibrotic role of IL-17A 31. An inverse relationship between Th17 cell number and the extent of skin fibrosis has been reported and it has been shown that IL-17A is able to induce MMP1 synthesis while simultaneously inhibiting type I collagen production in healthy and SSc fibroblasts 5,32. It has been found that IL-17A reduces the expression of alpha-smooth muscle actin induced by TGFβ in human fibroblasts and that the number of Th17 cells in skin of SSc patients inversely correlates with skin fibrosis 30; further, it has been described that IL-17A suppresses type I collagen synthesis in SSc fibroblasts 6.

In this report we found that PBMCs from SSc patients produce a significantly higher amount of IL- 17A compared to PBMCs from healthy subjects. Higher levels of IL-17A produced by PBMCs were associated to ILD and DU, whereas in patients with PAH the IL-17A production was significantly lower and no correlation between IL-17A production and mRSS was found. The findings concerning PAH, even if significant, should be interpreted with caution because of the limited number of patients; nevertheless these results are consistent with the assumption that the exact role of IL-17A in the pathogenesis of fibrosis is complex, and it would have rather a modulatory function than a definite pro-fibrotic or anti-fibrotic effect, depending on different tissues, disease stage and interaction with other cytokines that regulate fibrosis and vascular damage. It is possible that IL-17A may exerts a dual effect on fibrosis in the context of SSc, by acting in a different and often opposite way depending on its capacity to modulate the expression of various pro-fibrotic cytokines; interestingly in this study IL-17A production was significantly lower in SSc patients with PAH, leading to suppose that IL-17A could participate also in the modulation of physio-pathological processes involved in vascular disfunction. Likewise, the production of several pro-fibrotic cytokines, particularly FGF2, TGFβ and CTGF was significantly increased in PBMCs from SSc subjects compared to PBMCs from healthy subjects and was especially higher in patients with ILD compared to patients without ILD and in patients with DU.

One of the most interesting aspects shown by the results of this study is the opposite effect of IL- 17A inhibition on the expression of pro-fibrotic cytokines. In fact, while blocking IL-17A induces a significant reduction of FGF2 production, on the other hand it significantly increases the synthesis and expression of TGFβ and CTGF, and after IL-17A blockade, the TGFβ and CTGF production in cell cultures inversely correlated with IL-17A levels. Previous studies showed that IL-17A presents an antifibrotic effect that involves the inhibition of profibrotic genes regulated by TGFβ signalling; accordingly, in this study the neutralization of IL-17A is associated to an increase of TGβ, thus leading to hypothesize that IL-17A contributes to directly inhibit the production of the pro-fibrotic cytokine TGFβ. A recently published study provided evidence that conditioned-media from keratinocytes pre-treated simultaneously with IL-17A and TGFβ induces less collagen production in fibroblasts from SSc donors compared to those pre-treated with TGFβ only, and confirmed that IL-17A does not induce collagen deposition in human organotypic cultures 31. Additionally, the results of this study show that IL-17A neutralizing antibodies increase also the production of the pro-fibrotic cytokine CTGF. Several experimental models demonstrate that CTGF is involved in the pathogenesis of fibrogenic processes 33 and various studies show that it is highly expressed in serum, skin and lung tissues of SSc 34,35, but the modulation of this cytokine is not fully understood. Particularly there are few data concerning the pathogenic relationship between IL-17 and CTGF; accordingly with our results, it has been reported that IL-17A significantly decreases the mRNA expression of CTGF and α(Ι) collagen in scleroderma fibroblasts 6; conversely IL-17A addition in fibroblast cultures obtained from an animal model of bleomycin-induced fibrosis, increases the expression of CTGF and increases skin fibrosis 9.

Vitamin D insufficiency has been related to the development of many autoimmune diseases, including SSc 36,37. Low serum concentrations of 25(OH)D3 have been reported in SSc patients compared to healthy controls and several data indicate that in SSc patients poor vitamin D status is associated to a more aggressive disease and to various clinical manifestations, such as lung involvement, PAH and the extension of skin fibrosis 38-41. It has been shown that Vitamin D is able to modulate autoimmunity and inflammation and can interfere with fibrotic processes in several pathological conditions. Many studies suggested anti-fibrotic properties of Vitamin D; it has been reported that 1,25(OH)_2_D3 reduces the expression of pro-fibrotic factors, such as TGFβ and plasminogen activator, and the collagen synthesis in mesenchymal stem cells and, on the other hand, it stimulates the expression of anti-fibrotic factors, such as bone morphogenetic protein 7 and MMP-8 42. 1,25(OH)D3 can also inhibit the development of the pro-fibrotic phenotype in lung fibroblasts and epithelial cells 43 and can prevent the transformation of fibroblasts in myofibroblasts induced by TGF-β1 44.

The presented report shows that 1,25(OH)_2_D3 induces a significant reduction of IL-17A production from PBMCs both in SSc and in healthy subjects, in a dose dependent manner, accordingly with other previously published data demonstrating that 1,25(OH)_2_D3 has an inhibitory effect on Th17 cells and reduces the expression of IL17 both in SSc and in other autoimmune diseases 13, 45-49. Further, the presented data seem to confirm that 1,25(OH)_2_D3 can exert an inhibitory effect on the production of several pro-fibrotic cytokines, particularly TGFβ, CTGF and FGF2.

These results further support the suggestion of a potential anti-fibrotic effect of Vitamin D proved by various studies in several diseases, particularly in chronic liver disease, chronic kidney disease and lung fibrosis 50,51; nevertheless, despite a lot of studies showed a reduction of Vitamin D levels in SSc patients and a correlation of Vitamin D deficiency with several clinical manifestations of the disease, there are few data concerning the actual potential anti-fibrotic effects of 1,25(OH)_2_D3 in SSc. Only one previously published experimental study showed that the activation of Vitamin D Receptor (VDR) by the synthetic VDR agonist paracalcitiol reduces the stimulatory effects of TGFβ on collagen production and on the formation of stress fibers in SSc fibroblasts, and prevents experimental fibrosis in two different mouse models of SSc 52. The results of this study are consistent with a potential inhibitory effect of 1,25(OH)_2_D3 on profibrotic cytokine production by immune cells and on IL-17A which is in turn involved in the regulation of pro-fibrotic cytokines.

This study presents some limitations. Cytokine production has been evaluated *in vitro* in PBMCs, whereas *in vivo* also other cell types, both in peripheral blood and at tissue level, secrete cytokines and participate to the pathogenesis of tissue and vascular changes typical of SSc. Nevertheless, lymphocytes and monocytes, that represent the majority of the PBMCs population, are among the main effectors of the pathogenic events that lead to fibrosis and vascular damage, playing a central role in the pathogenesis of the disease. Another limitation of the study is represented by the high concentrations of 1,25(OH)_2_D3 used in PBMCs cultures and the 1,25(OH)_2_D3 effects on cytokine production that are observed, for some pro-fibrotic cytokines, only at supraphysiological concentrations. Actually, the evaluation of the effects of 1,25(OH)_2_D3 in immune cells *in vitro* is an artificial condition and the high concentrations employed are not the same achievable in human peripheral blood *in vivo*. Nevertheless, higher concentrations of 1,25(OH)_2_D3 could be reached locally at sites of inflammation, where several activated immune cells can produce themselves Vitamin D metabolites 53. Thus, the physio-pathological relevance of the *in vitro* observations can be reasonable, even if the translation to human interventions with Vitamin D supplementation is difficult. The inhibitory effect of 1,25(OH)_2_D3 on the production of IL-17A and the pro-fibrotic cytokines could be overinterpreted owing to the overall inhibition of T-cell activation induced by 25(OH)_2_D3. However, this effect had never been shown before in SSc and on these specific cytokines.

## Conclusions

Despite many evidences appear to confirm the involvement of IL-17 in physio-pathological processes in SSc, its exact role in this disease remains uncertain and the clinical implications of the altered IL-17 profile are still controversial, even if IL-17 could represent an intriguing potential therapeutic target. The different and often opposite data concerning IL-17A levels and the relationship with pro-fibrotic cytokines and fibrotic processes observed in several tissues of SSc patients, would suggest that IL-17A modulates fibrogenesis rather than exerts a net pro-fibrotic or anti-fibrotic effect. Thus, *in vivo* investigations would be needed to evaluate the real role of IL- 17A in the development of fibrotic and vascular clinical findings in SSc, and to assess if the anti-IL- 17A agents in clinical practice would be beneficial or detrimental.

1,25(OH)_2_D3 inhibits both IL-17A and pro-fibrotic cytokines, confirming its potential anti-fibrotic effect and supporting the hypothesis that supplementation of Vitamin D in SSc patients could have a potentially mitigating effect on fibrotic processes.

## Figures and Tables

**Figure 5:**
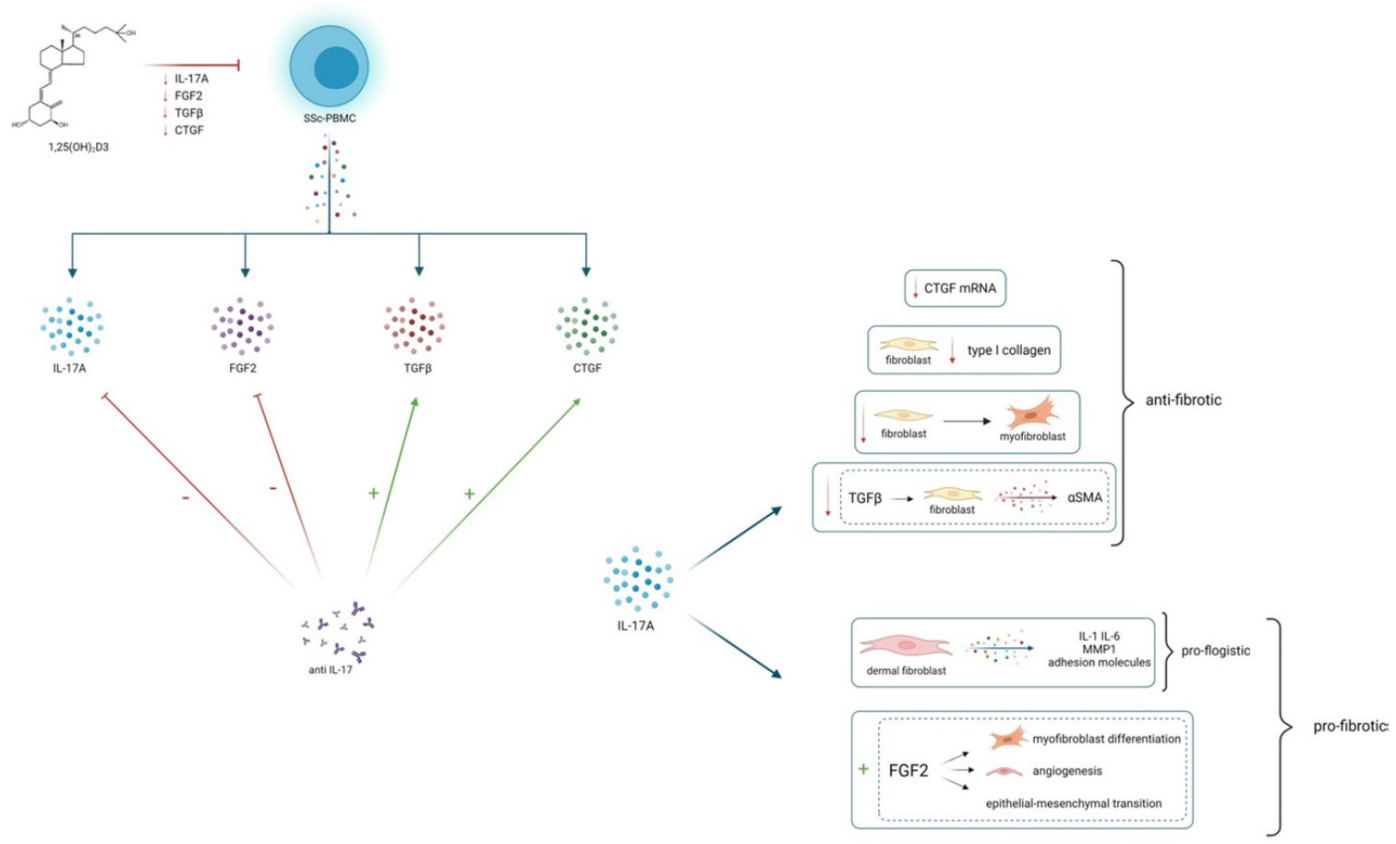
25(OH)_2_D3 reduces *in vitro* the production of IL-17A and several pro-fibrotic cytokines, which are in turn modulated by IL-17A. This could lead to hypothesize an overall anti-fibrotic effect of 25(OH)_2_D3. IL-17A can play a modulatory role on fibrogenesis, by stimulating or inhibiting several pro-fibrotic cytokines, probably depending on the interaction with immune cells and other cytokines whose production can vary in different tissues, in distinct disease phenotypes and in different stages of disease

**Table 1:** Clinical and demographic characteristics of SSc patients

Characteristics	SSc (n=51)	Healthy (n=31)
Age (years) mean ± SD	57.64±11.75	53.12±13.08
Gender female n(%)	47 (92.15%)	27 (87%)
Disease duration (years) mean ± SD	14.58±8.97	-
ILD n(%)	25 (49%)	-
PAH n(%)	7 (13.7%)	-
DU n(%)	33 (64.7%)	-
Esopaheal dysfunction n(%)	36 (70.5%)	-
Gastrointestinal involvement	24 (47%)	-
Arthritis n(%)	16 (31.37%)	-
Rodnan skin score mean ± SD	19.39±9.19	-
Positive ANA n(%)	44 (86.27)	-
Positive Anti Centromere n(%)	19 (37.25%)	-
Positive Anti s Scl-70 n(%)	27 (52.9%)	-
25(OH) Vitamin D serum levels (ng/ml) mean ±SD	15.35±6.2	18.08±5.03

**Table 2:** Cytokine production by PBMCs from healthy donors and SSc subjects

Cytokines	Healthy	SSc	p
IL-17A (pg/ml ± SD)	150.8±57.3	464.5±139.05	p<0.001
FGF2 (pg/ml ± SD)	8.9±1.5	15.77±5.18	p<0.01
TGFβ (pg/ml ± SD)	663.7±84.9	784.1±239,6	p<0.01
CTGF (pg/ml ± SD)	80.1±11.4	105.2±14.1	p<0.001

## Abbreviations

SSc: systemic sclerosis
1,25(OH)_2_D3: 1,25(OH)_2_VitaminD3
PBMCs: peripheral blood mononuclear cells
mRSS: modified Rodnan skin score
ANA: anti-nuclear antibodies
ACAs: anti- centromeric antibodies
anti-ENA: anti-Extractable Nuclear Antigens
HRP: horseradish peroxidase
TMB: 3,3′,5,5′-tetramethylbenzidine
ANOVA: analysis of variance
ILD: interstitial lung disease
PAH: pulmonary arterial hypertension
DU: digital ulcers
VDR: Vitamin D Receptor
